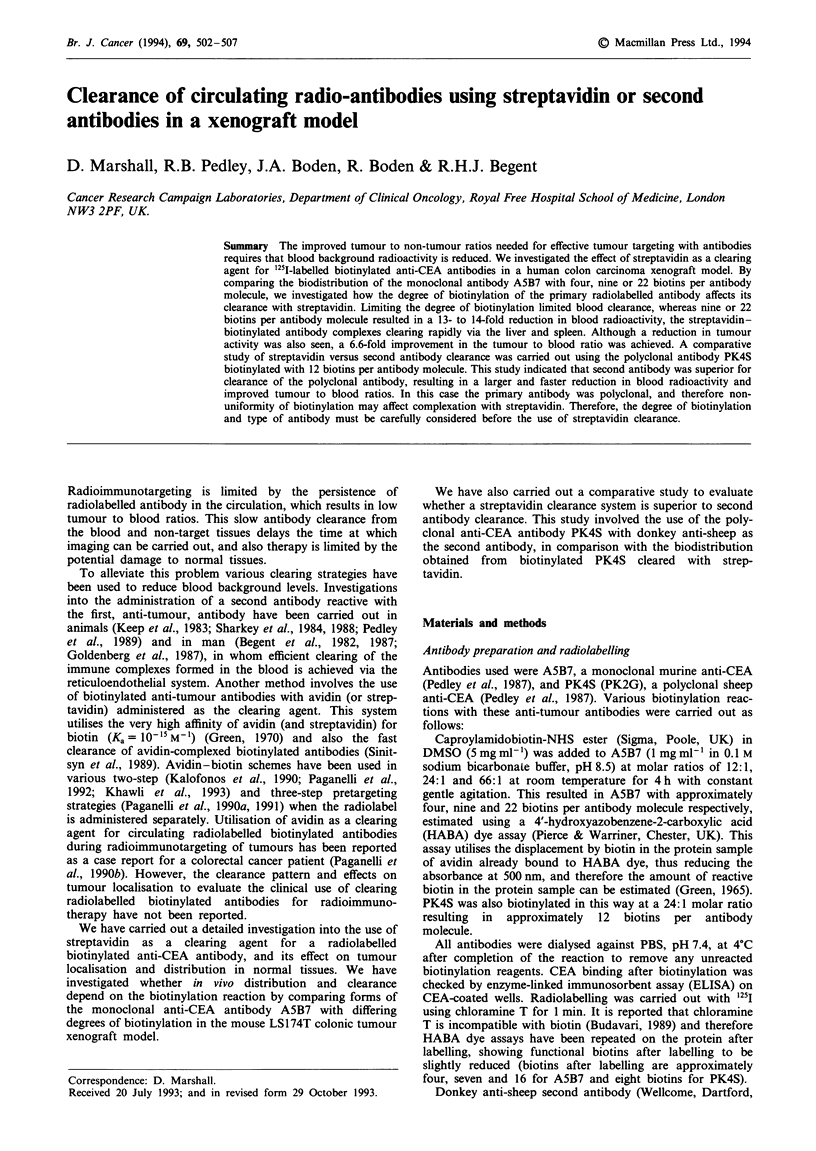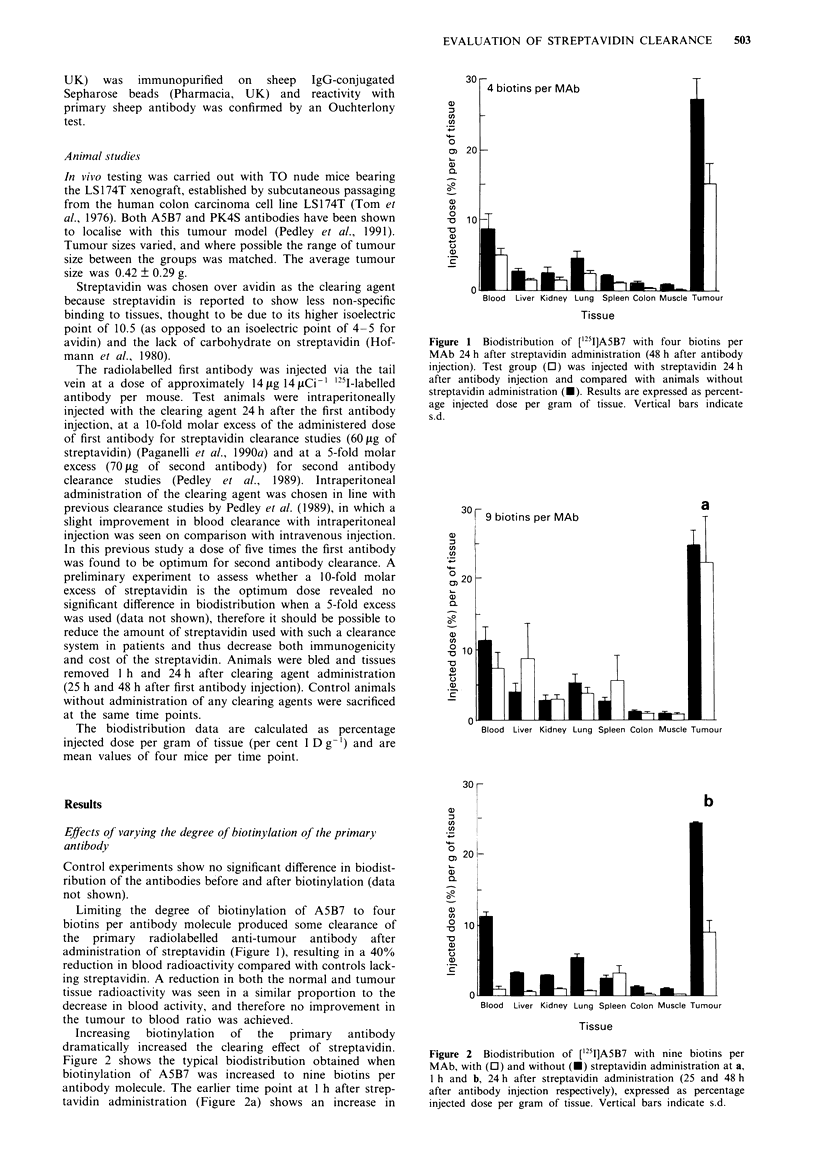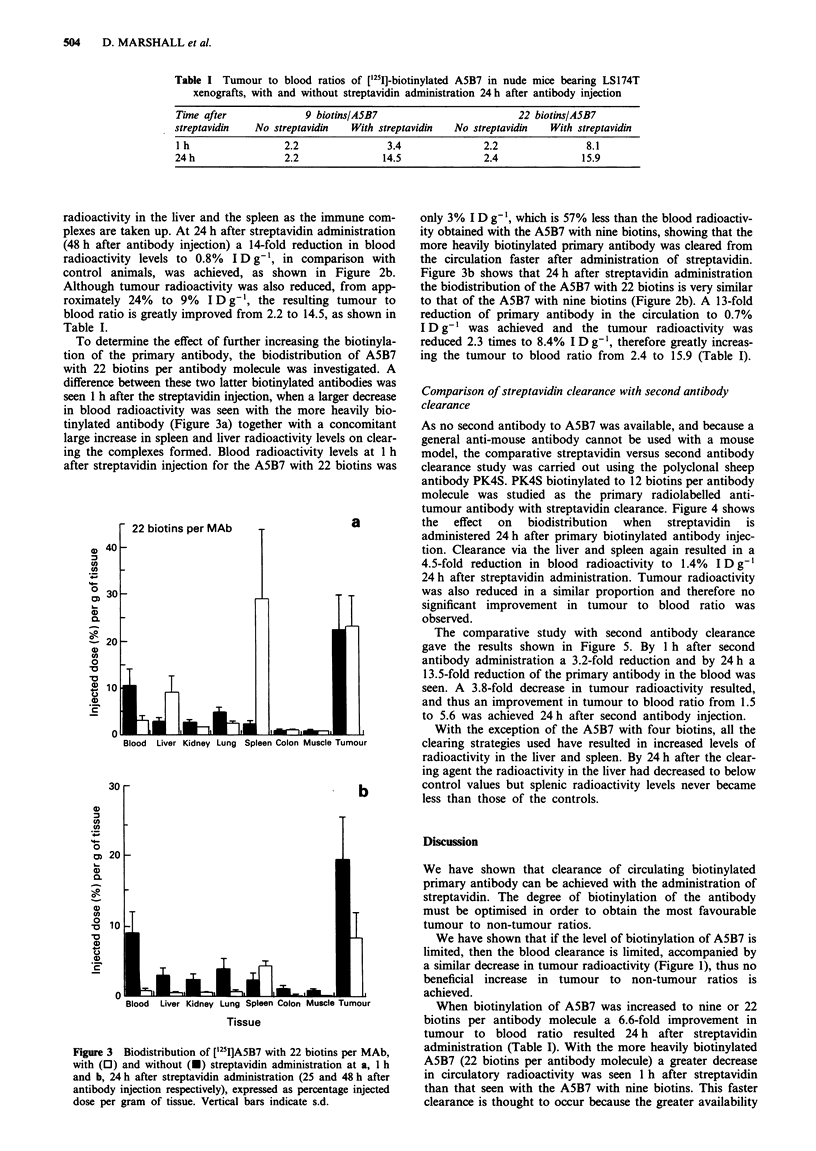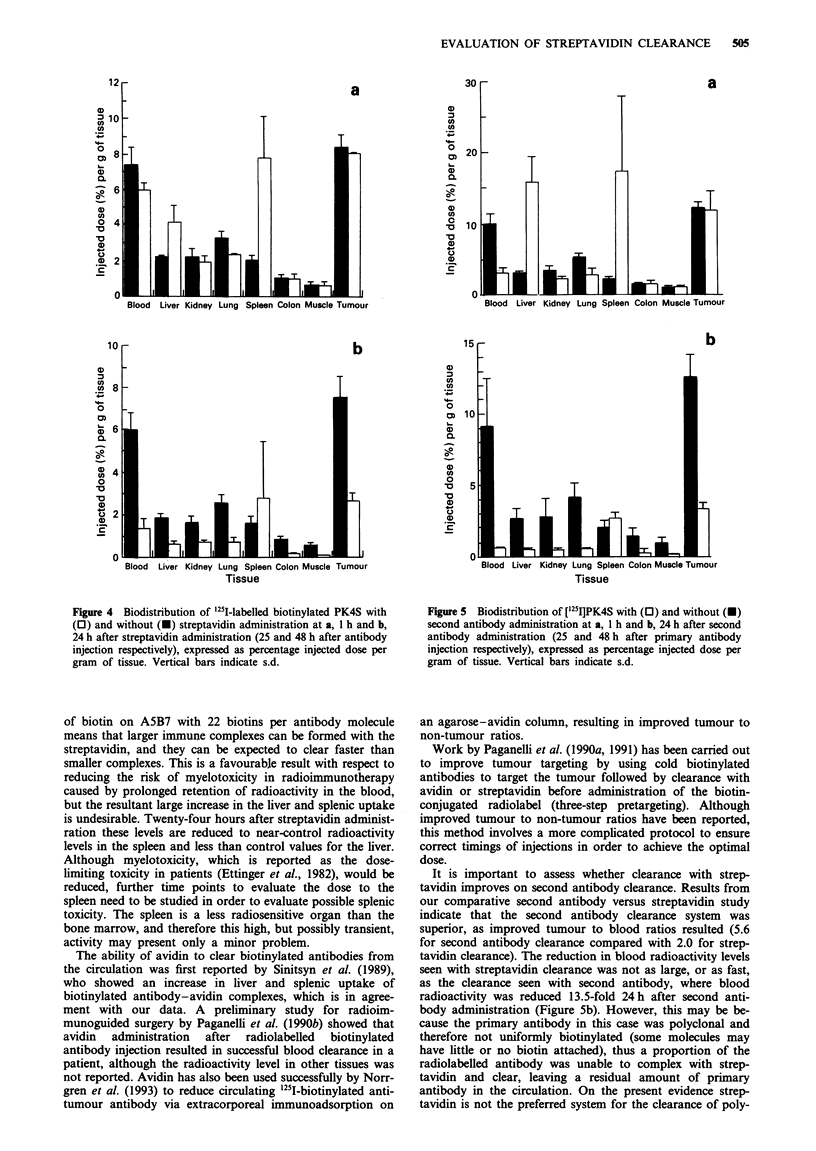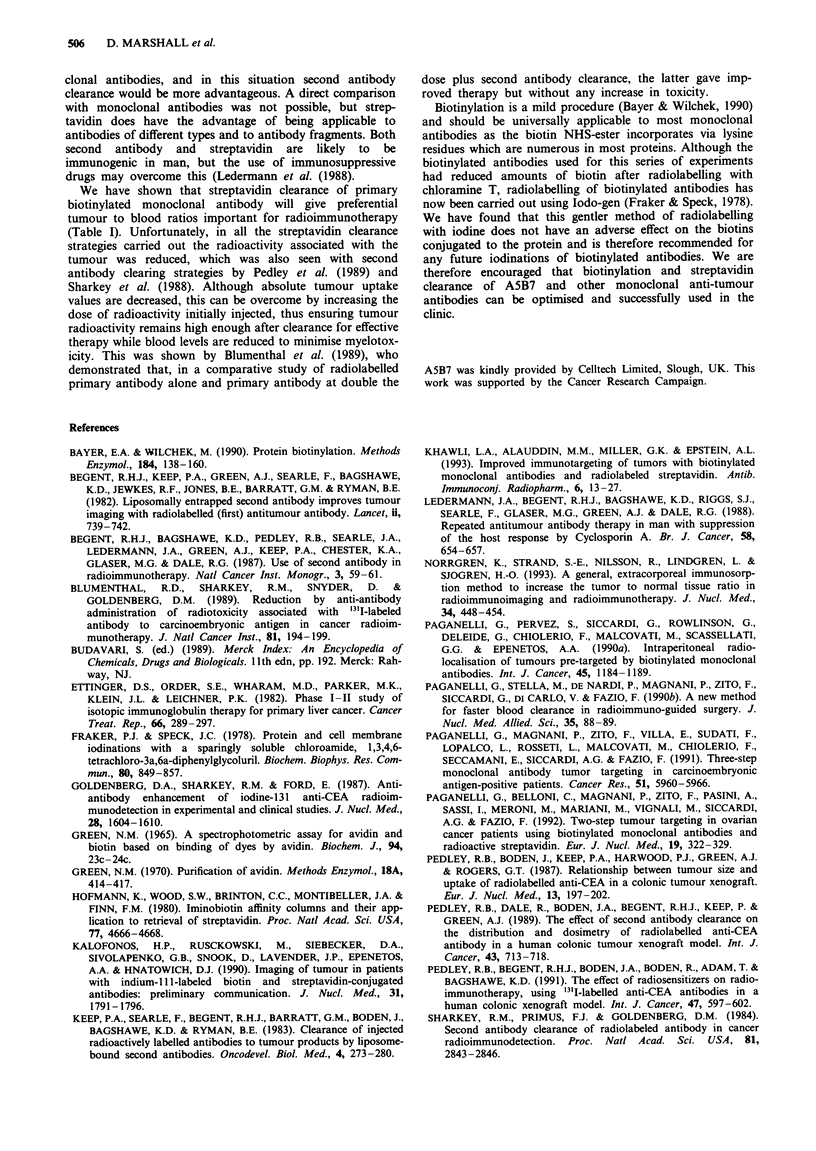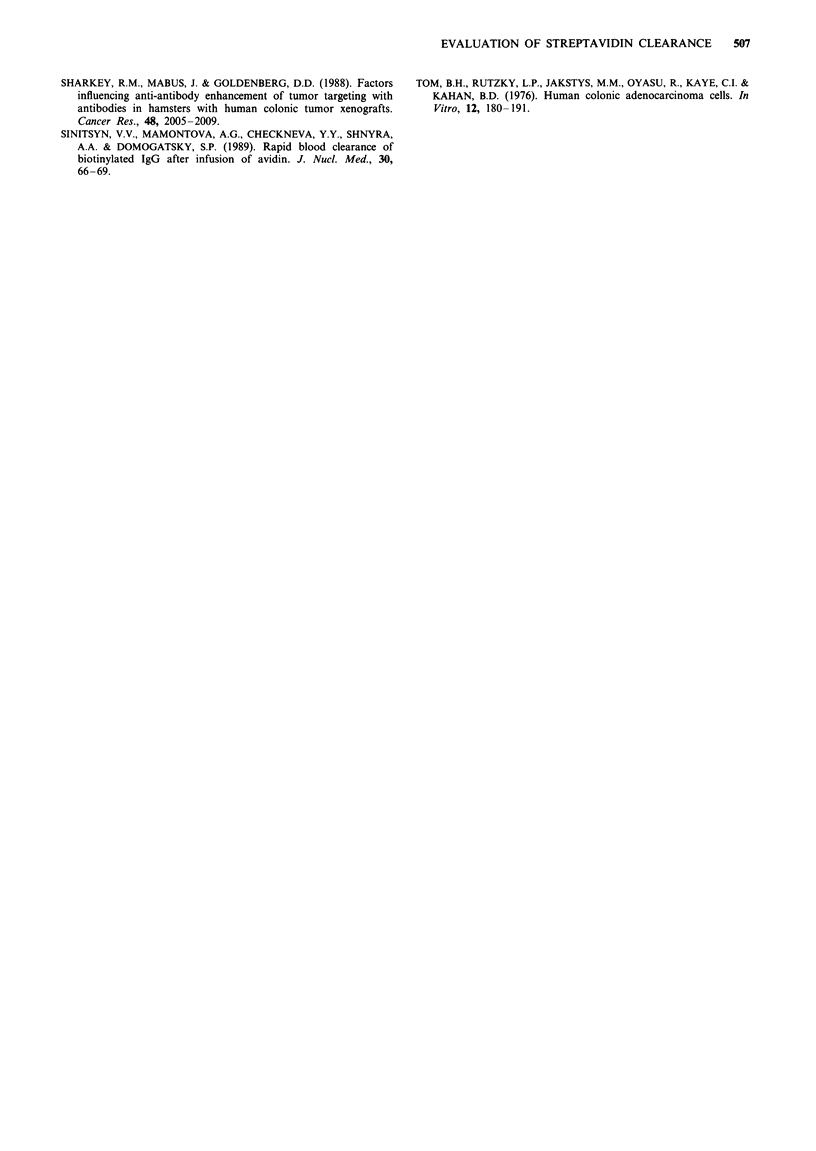# Clearance of circulating radio-antibodies using streptavidin or second antibodies in a xenograft model.

**DOI:** 10.1038/bjc.1994.91

**Published:** 1994-03

**Authors:** D. Marshall, R. B. Pedley, J. A. Boden, R. Boden, R. H. Begent

**Affiliations:** Department of Clinical Oncology, Royal Free Hospital School of Medicine, London, UK.

## Abstract

The improved tumour to non-tumour ratios needed for effective tumour targeting with antibodies requires that blood background radioactivity is reduced. We investigated the effect of streptavidin as a clearing agent for 125I-labelled biotinylated anti-CEA antibodies in a human colon carcinoma xenograft model. By comparing the biodistribution of the monoclonal antibody A5B7 with four, nine or 22 biotins per antibody molecule, we investigated how the degree of biotinylation of the primary radiolabelled antibody affects its clearance with streptavidin. Limiting the degree of biotinylation limited blood clearance, whereas nine or 22 biotins per antibody molecule resulted in a 13- to 14-fold reduction in blood radioactivity, the streptavidin-biotinylated antibody complexes clearing rapidly via the liver and spleen. Although a reduction in tumour activity was also seen, a 6.6-fold improvement in the tumour to blood ratio was achieved. A comparative study of streptavidin versus second antibody clearance was carried out using the polyclonal antibody PK4S biotinylated with 12 biotins per antibody molecule. This study indicated that second antibody was superior for clearance of the polyclonal antibody, resulting in a larger and faster reduction in blood radioactivity and improved tumour to blood ratios. In this case the primary antibody was polyclonal, and therefore non-uniformity of biotinylation may affect complexation with streptavidin. Therefore, the degree of biotinylation and type of antibody must be carefully considered before the use of streptavidin clearance.


Br. J. Cancer (1994), 69, 502–507 © Macmillan Press Ltd., 1994

### Clearance of circulating radio-antibodies using streptavidin or second antibodies in a xenograft model

D. Marshall, R.B. Pedley, J.A. Boden, R. Boden & R.H.J. Begent

*Cancer Research Campaign Laboratories, Department of Clinical Oncology, Royal Free Hospital School of Medicine, London NW3 2PF, UK.*

**Summary** The improved tumour to non-tumour ratios needed for effective tumour targeting with antibodies requires that blood background radioactivity is reduced. We investigated the effect of streptavidin as a clearing agent for $^{125}$I-labelled biotinylated anti-CEA antibodies in a human colon carcinoma xenograft model. By comparing the biodistribution of the monoclonal antibody A5B7 with four, nine or 22 biotins per antibody molecule, we investigated how the degree of biotinylation of the primary radiolabelled antibody affects its clearance with streptavidin. Limiting the degree of biotinylation limited blood clearance, whereas nine or 22 biotins per antibody molecule resulted in a 13- to 14-fold reduction in blood radioactivity, the streptavidin–biotinylated antibody complexes clearing rapidly via the liver and spleen. Although a reduction in tumour activity was also seen, a 6.6-fold improvement in the tumour to blood ratio was achieved. A comparative study of streptavidin versus second antibody clearance was carried out using the polyclonal antibody PK4S biotinylated with 12 biotins per antibody molecule. This study indicated that second antibody was superior for clearance of the polyclonal antibody, resulting in a larger and faster reduction in blood radioactivity and improved tumour to blood ratios. In this case the primary antibody was polyclonal, and therefore non-uniformity of biotinylation may affect complexation with streptavidin. Therefore, the degree of biotinylation and type of antibody must be carefully considered before the use of streptavidin clearance.

Radioimmunotargeting is limited by the persistence of radiolabelled antibody in the circulation, which results in low tumour to blood ratios. This slow antibody clearance from the blood and non-target tissues delays the time at which imaging can be carried out, and also therapy is limited by the potential damage to normal tissues.

To alleviate this problem various clearing strategies have been used to reduce blood background levels. Investigations into the administration of a second antibody reactive with the first, anti-tumour, antibody have been carried out in animals (Keep *et al.*, 1983; Sharkey *et al.*, 1984, 1988; Pedley *et al.*, 1989) and in man (Begent *et al.*, 1982, 1987; Goldenberg *et al.*, 1987), in whom efficient clearing of the immune complexes formed in the blood is achieved via the reticuloendothelial system. Another method involves the use of biotinylated anti-tumour antibodies with avidin (or streptavidin) administered as the clearing agent. This system utilises the very high affinity of avidin (and streptavidin) for biotin ($K_a = 10^{-15}\,\mathrm{M}^{-1}$) (Green, 1970) and also the fast clearance of avidin-complexed biotinylated antibodies (Sinitsyn *et al.*, 1989). Avidin–biotin schemes have been used in various two-step (Kalofonos *et al.*, 1990; Paganelli *et al.*, 1992; Khawli *et al.*, 1993) and three-step pretargeting strategies (Paganelli *et al.*, 1990*a*, 1991) when the radiolabel is administered separately. Utilisation of avidin as a clearing agent for circulating radiolabelled biotinylated antibodies during radioimmunotargeting of tumours has been reported as a case report for a colorectal cancer patient (Paganelli *et al.*, 1990*b*). However, the clearance pattern and effects on tumour localisation to evaluate the clinical use of clearing radiolabelled biotinylated antibodies for radioimmunotherapy have not been reported.

We have carried out a detailed investigation into the use of streptavidin as a clearing agent for a radiolabelled biotinylated anti-CEA antibody, and its effect on tumour localisation and distribution in normal tissues. We have investigated whether *in vivo* distribution and clearance depend on the biotinylation reaction by comparing forms of the monoclonal anti-CEA antibody A5B7 with differing degrees of biotinylation in the mouse LS174T colonic tumour xenograft model.

Correspondence: D. Marshall.
Received 20 July 1993; and in revised form 29 October 1993.

We have also carried out a comparative study to evaluate whether a streptavidin clearance system is superior to second antibody clearance. This study involved the use of the polyclonal anti-CEA antibody PK4S with donkey anti-sheep as the second antibody, in comparison with the biodistribution obtained from biotinylated PK4S cleared with streptavidin.

### Materials and methods

### *Antibody preparation and radiolabelling*

Antibodies used were A5B7, a monoclonal murine anti-CEA (Pedley *et al.*, 1987), and PK4S (PK2G), a polyclonal sheep anti-CEA (Pedley *et al.*, 1987). Various biotinylation reactions with these anti-tumour antibodies were carried out as follows:

Caproylamidobiotin-NHS ester (Sigma, Poole, UK) in DMSO (5 mg ml$^{-1}$) was added to A5B7 (1 mg ml$^{-1}$ in 0.1 M sodium bicarbonate buffer, pH 8.5) at molar ratios of 12:1, 24:1 and 66:1 at room temperature for 4 h with constant gentle agitation. This resulted in A5B7 with approximately four, nine and 22 biotins per antibody molecule respectively, estimated using a 4′-hydroxyazobenzene-2-carboxylic acid (HABA) dye assay (Pierce & Warriner, Chester, UK). This assay utilises the displacement by biotin in the protein sample of avidin already bound to HABA dye, thus reducing the absorbance at 500 nm, and therefore the amount of reactive biotin in the protein sample can be estimated (Green, 1965). PK4S was also biotinylated in this way at a 24:1 molar ratio resulting in approximately 12 biotins per antibody molecule.

All antibodies were dialysed against PBS, pH 7.4, at 4°C after completion of the reaction to remove any unreacted biotinylation reagents. CEA binding after biotinylation was checked by enzyme-linked immunosorbent assay (ELISA) on CEA-coated wells. Radiolabelling was carried out with $^{125}$I using chloramine T for 1 min. It is reported that chloramine T is incompatible with biotin (Budavari, 1989) and therefore HABA dye assays have been repeated on the protein after labelling, showing functional biotins after labelling to be slightly reduced (biotins after labelling are approximately four, seven and 16 for A5B7 and eight biotins for PK4S).

Donkey anti-sheep second antibody (Wellcome, Dartford,

UK) was immunopurified on sheep IgG-conjugated Sepharose beads (Pharmacia, UK) and reactivity with primary sheep antibody was confirmed by an Ouchterlony test.

*Animal studies*

*In vivo* testing was carried out with TO nude mice bearing the LS174T xenograft, established by subcutaneous passaging from the human colon carcinoma cell line LS174T (Tom *et al.*, 1976). Both A5B7 and PK4S antibodies have been shown to localise with this tumour model (Pedley *et al.*, 1991). Tumour sizes varied, and where possible the range of tumour size between the groups was matched. The average tumour size was 0.42 ± 0.29 g.

Streptavidin was chosen over avidin as the clearing agent because streptavidin is reported to show less non-specific binding to tissues, thought to be due to its higher isoelectric point of 10.5 (as opposed to an isoelectric point of 4–5 for avidin) and the lack of carbohydrate on streptavidin (Hofmann *et al.*, 1980).

The radiolabelled first antibody was injected via the tail vein at a dose of approximately 14 μg 14 μCi$^{-1}$ $^{125}$I-labelled antibody per mouse. Test animals were intraperitoneally injected with the clearing agent 24 h after the first antibody injection, at a 10-fold molar excess of the administered dose of first antibody for streptavidin clearance studies (60 μg of streptavidin) (Paganelli *et al.*, 1990*a*) and at a 5-fold molar excess (70 μg of second antibody) for second antibody clearance studies (Pedley *et al.*, 1989). Intraperitoneal administration of the clearing agent was chosen in line with previous clearance studies by Pedley *et al.* (1989), in which a slight improvement in blood clearance with intraperitoneal injection was seen on comparison with intravenous injection. In this previous study a dose of five times the first antibody was found to be optimum for second antibody clearance. A preliminary experiment to assess whether a 10-fold molar excess of streptavidin is the optimum dose revealed no significant difference in biodistribution when a 5-fold excess was used (data not shown), therefore it should be possible to reduce the amount of streptavidin used with such a clearance system in patients and thus decrease both immunogenicity and cost of the streptavidin. Animals were bled and tissues removed 1 h and 24 h after clearing agent administration (25 h and 48 h after first antibody injection). Control animals without administration of any clearing agents were sacrificed at the same time points.

The biodistribution data are calculated as percentage injected dose per gram of tissue (per cent I D g$^{-1}$) and are mean values of four mice per time point.

### Results

*Effects of varying the degree of biotinylation of the primary antibody*

Control experiments show no significant difference in biodistribution of the antibodies before and after biotinylation (data not shown).

Limiting the degree of biotinylation of A5B7 to four biotins per antibody molecule produced some clearance of the primary radiolabelled anti-tumour antibody after administration of streptavidin (Figure 1), resulting in a 40% reduction in blood radioactivity compared with controls lacking streptavidin. A reduction in both the normal and tumour tissue radioactivity was seen in a similar proportion to the decrease in blood activity, and therefore no improvement in the tumour to blood ratio was achieved.

Increasing biotinylation of the primary antibody dramatically increased the clearing effect of streptavidin. Figure 2 shows the typical biodistribution obtained when biotinylation of A5B7 was increased to nine biotins per antibody molecule. The earlier time point at 1 h after streptavidin administration (Figure 2a) shows an increase in

**Figure 1** Biodistribution of [$^{125}$I]A5B7 with four biotins per MAb 24 h after streptavidin administration (48 h after antibody injection). Test group (□) was injected with streptavidin 24 h after antibody injection and compared with animals without streptavidin administration (■). Results are expressed as percentage injected dose per gram of tissue. Vertical bars indicate s.d.

**Figure 2** Biodistribution of [$^{125}$I]A5B7 with nine biotins per MAb, with (□) and without (■) streptavidin administration at **a**, 1 h and **b**, 24 h after streptavidin administration (25 and 48 h after antibody injection respectively), expressed as percentage injected dose per gram of tissue. Vertical bars indicate s.d.

**Table I** Tumour to blood ratios of [$^{125}$I]-biotinylated A5B7 in nude mice bearing LS174T xenografts, with and without streptavidin administration 24 h after antibody injection

| *Time after streptavidin* | *9 biotins/A5B7* | | *22 biotins/A5B7* | |
|---|---|---|---|---|
| | *No streptavidin* | *With streptavidin* | *No streptavidin* | *With streptavidin* |
| 1 h | 2.2 | 3.4 | 2.2 | 8.1 |
| 24 h | 2.2 | 14.5 | 2.4 | 15.9 |

radioactivity in the liver and the spleen as the immune complexes are taken up. At 24 h after streptavidin administration (48 h after antibody injection) a 14-fold reduction in blood radioactivity levels to 0.8% I D $g^{-1}$, in comparison with control animals, was achieved, as shown in Figure 2b. Although tumour radioactivity was also reduced, from approximately 24% to 9% I D $g^{-1}$, the resulting tumour to blood ratio is greatly improved from 2.2 to 14.5, as shown in Table I.

To determine the effect of further increasing the biotinylation of the primary antibody, the biodistribution of A5B7 with 22 biotins per antibody molecule was investigated. A difference between these two latter biotinylated antibodies was seen 1 h after the streptavidin injection, when a larger decrease in blood radioactivity was seen with the more heavily biotinylated antibody (Figure 3a) together with a concomitant large increase in spleen and liver radioactivity levels on clearing the complexes formed. Blood radioactivity levels at 1 h after streptavidin injection for the A5B7 with 22 biotins was

**Figure 3** Biodistribution of [$^{125}$I]A5B7 with 22 biotins per MAb, with (□) and without (■) streptavidin administration at **a**, 1 h and **b**, 24 h after streptavidin administration (25 and 48 h after antibody injection respectively), expressed as percentage injected dose per gram of tissue. Vertical bars indicate s.d.

only 3% I D $g^{-1}$, which is 57% less than the blood radioactivity obtained with the A5B7 with nine biotins, showing that the more heavily biotinylated primary antibody was cleared from the circulation faster after administration of streptavidin. Figure 3b shows that 24 h after streptavidin administration the biodistribution of the A5B7 with 22 biotins is very similar to that of the A5B7 with nine biotins (Figure 2b). A 13-fold reduction of primary antibody in the circulation to 0.7% I D $g^{-1}$ was achieved and the tumour radioactivity was reduced 2.3 times to 8.4% I D $g^{-1}$, therefore greatly increasing the tumour to blood ratio from 2.4 to 15.9 (Table I).

### *Comparison of streptavidin clearance with second antibody clearance*

As no second antibody to A5B7 was available, and because a general anti-mouse antibody cannot be used with a mouse model, the comparative streptavidin versus second antibody clearance study was carried out using the polyclonal sheep antibody PK4S. PK4S biotinylated to 12 biotins per antibody molecule was studied as the primary radiolabelled anti-tumour antibody with streptavidin clearance. Figure 4 shows the effect on biodistribution when streptavidin is administered 24 h after primary biotinylated antibody injection. Clearance via the liver and spleen again resulted in a 4.5-fold reduction in blood radioactivity to 1.4% I D $g^{-1}$ 24 h after streptavidin administration. Tumour radioactivity was also reduced in a similar proportion and therefore no significant improvement in tumour to blood ratio was observed.

The comparative study with second antibody clearance gave the results shown in Figure 5. By 1 h after second antibody administration a 3.2-fold reduction and by 24 h a 13.5-fold reduction of the primary antibody in the blood was seen. A 3.8-fold decrease in tumour radioactivity resulted, and thus an improvement in tumour to blood ratio from 1.5 to 5.6 was achieved 24 h after second antibody injection.

With the exception of the A5B7 with four biotins, all the clearing strategies used have resulted in increased levels of radioactivity in the liver and spleen. By 24 h after the clearing agent the radioactivity in the liver had decreased to below control values but splenic radioactivity levels never became less than those of the controls.

### Discussion

We have shown that clearance of circulating biotinylated primary antibody can be achieved with the administration of streptavidin. The degree of biotinylation of the antibody must be optimised in order to obtain the most favourable tumour to non-tumour ratios.

We have shown that if the level of biotinylation of A5B7 is limited, then the blood clearance is limited, accompanied by a similar decrease in tumour radioactivity (Figure 1), thus no beneficial increase in tumour to non-tumour ratios is achieved.

When biotinylation of A5B7 was increased to nine or 22 biotins per antibody molecule a 6.6-fold improvement in tumour to blood ratio resulted 24 h after streptavidin administration (Table I). With the more heavily biotinylated A5B7 (22 biotins per antibody molecule) a greater decrease in circulatory radioactivity was seen 1 h after streptavidin than that seen with the A5B7 with nine biotins. This faster clearance is thought to occur because the greater availability

**Figure 4** Biodistribution of $^{125}$I-labelled biotinylated PK4S with (□) and without (■) streptavidin administration at **a**, 1 h and **b**, 24 h after streptavidin administration (25 and 48 h after antibody injection respectively), expressed as percentage injected dose per gram of tissue. Vertical bars indicate s.d.

**Figure 5** Biodistribution of [$^{125}$I]PK4S with (□) and without (■) second antibody administration at **a**, 1 h and **b**, 24 h after second antibody administration (25 and 48 h after primary antibody injection respectively), expressed as percentage injected dose per gram of tissue. Vertical bars indicate s.d.

of biotin on A5B7 with 22 biotins per antibody molecule means that larger immune complexes can be formed with the streptavidin, and they can be expected to clear faster than smaller complexes. This is a favourable result with respect to reducing the risk of myelotoxicity in radioimmunotherapy caused by prolonged retention of radioactivity in the blood, but the resultant large increase in the liver and splenic uptake is undesirable. Twenty-four hours after streptavidin administration these levels are reduced to near-control radioactivity levels in the spleen and less than control values for the liver. Although myelotoxicity, which is reported as the dose-limiting toxicity in patients (Ettinger *et al.*, 1982), would be reduced, further time points to evaluate the dose to the spleen need to be studied in order to evaluate possible splenic toxicity. The spleen is a less radiosensitive organ than the bone marrow, and therefore this high, but possibly transient, activity may present only a minor problem.

The ability of avidin to clear biotinylated antibodies from the circulation was first reported by Sinitsyn *et al.* (1989), who showed an increase in liver and splenic uptake of biotinylated antibody–avidin complexes, which is in agreement with our data. A preliminary study for radioimmunoguided surgery by Paganelli *et al.* (1990*b*) showed that avidin administration after radiolabelled biotinylated antibody injection resulted in successful blood clearance in a patient, although the radioactivity level in other tissues was not reported. Avidin has also been used successfully by Norrgren *et al.* (1993) to reduce circulating $^{125}$I-biotinylated anti-tumour antibody via extracorporeal immunoadsorption on an agarose–avidin column, resulting in improved tumour to non-tumour ratios.

Work by Paganelli *et al.* (1990*a*, 1991) has been carried out to improve tumour targeting by using cold biotinylated antibodies to target the tumour followed by clearance with avidin or streptavidin before administration of the biotin-conjugated radiolabel (three-step pretargeting). Although improved tumour to non-tumour ratios have been reported, this method involves a more complicated protocol to ensure correct timings of injections in order to achieve the optimal dose.

It is important to assess whether clearance with streptavidin improves on second antibody clearance. Results from our comparative second antibody versus streptavidin study indicate that the second antibody clearance system was superior, as improved tumour to blood ratios resulted (5.6 for second antibody clearance compared with 2.0 for streptavidin clearance). The reduction in blood radioactivity levels seen with streptavidin clearance was not as large, or as fast, as the clearance seen with second antibody, where blood radioactivity was reduced 13.5-fold 24 h after second antibody administration (Figure 5b). However, this may be because the primary antibody in this case was polyclonal and therefore not uniformly biotinylated (some molecules may have little or no biotin attached), thus a proportion of the radiolabelled antibody was unable to complex with streptavidin and clear, leaving a residual amount of primary antibody in the circulation. On the present evidence streptavidin is not the preferred system for the clearance of polyclonal antibodies, and in this situation second antibody clearance would be more advantageous. A direct comparison with monoclonal antibodies was not possible, but streptavidin does have the advantage of being applicable to antibodies of different types and to antibody fragments. Both second antibody and streptavidin are likely to be immunogenic in man, but the use of immunosuppressive drugs may overcome this (Ledermann et al. (1988).

We have shown that streptavidin clearance of primary biotinylated monoclonal antibody will give preferential tumour to blood ratios important for radioimmunotherapy (Table I). Unfortunately, in all the streptavidin clearance strategies carried out the radioactivity associated with the tumour was reduced, which was also seen with second antibody clearing strategies by Pedley et al. (1989) and Sharkey et al. (1988). Although absolute tumour uptake values are decreased, this can be overcome by increasing the dose of radioactivity initially injected, thus ensuring tumour radioactivity remains high enough after clearance for effective therapy while blood levels are reduced to minimise myelotoxicity. This was shown by Blumenthal et al. (1989), who demonstrated that, in a comparative study of radiolabelled primary antibody alone and primary antibody at double the dose plus second antibody clearance, the latter gave improved therapy but without any increase in toxicity.

Biotinylation is a mild procedure (Bayer & Wilchek, 1990) and should be universally applicable to most monoclonal antibodies as the biotin NHS-ester incorporates via lysine residues which are numerous in most proteins. Although the biotinylated antibodies used for this series of experiments had reduced amounts of biotin after radiolabelling with chloramine T, radiolabelling of biotinylated antibodies has now been carried out using Iodo-gen (Fraker & Speck, 1978). We have found that this gentler method of radiolabelling with iodine does not have an adverse effect on the biotins conjugated to the protein and is therefore recommended for any future iodinations of biotinylated antibodies. We are therefore encouraged that biotinylation and streptavidin clearance of A5B7 and other monoclonal anti-tumour antibodies can be optimised and successfully used in the clinic.

A5B7 was kindly provided by Celltech Limited, Slough, UK. This work was supported by the Cancer Research Campaign.

### References

BAYER, E.A. & WILCHEK, M. (1990). Protein biotinylation. *Methods Enzymol.*, **184**, 138-160.

BEGENT, R.H.J., KEEP, P.A., GREEN, A.J., SEARLE, F., BAGSHAWE, K.D., JEWKES, R.F., JONES, B.E., BARRATT, G.M. & RYMAN, B.E. (1982). Liposomally entrapped second antibody improves tumour imaging with radiolabelled (first) antitumour antibody. *Lancet*, **ii**, 739-742.

BEGENT, R.H.J., BAGSHAWE, K.D., PEDLEY, R.B., SEARLE, J.A., LEDERMANN, J.A., GREEN, A.J., KEEP, P.A., CHESTER, K.A., GLASER, M.G. & DALE, R.G. (1987). Use of second antibody in radioimmunotherapy. *Natl Cancer Inst. Monogr.*, **3**, 59-61.

BLUMENTHAL, R.D., SHARKEY, R.M., SNYDER, D. & GOLDENBERG, D.M. (1989). Reduction by anti-antibody administration of radiotoxicity associated with $^{131}$I-labeled antibody to carcinoembryonic antigen in cancer radioimmunotherapy. *J. Natl Cancer Inst.*, **81**, 194-199.

BUDAVARI, S. (ed.) (1989). *Merck Index: An Encyclopedia of Chemicals, Drugs and Biologicals*. 11th edn, pp. 192. Merck: Rahway, NJ.

ETTINGER, D.S., ORDER, S.E., WHARAM, M.D., PARKER, M.K., KLEIN, J.L. & LEICHNER, P.K. (1982). Phase I-II study of isotopic immunoglobulin therapy for primary liver cancer. *Cancer Treat. Rep.*, **66**, 289-297.

FRAKER, P.J. & SPECK, J.C. (1978). Protein and cell membrane iodinations with a sparingly soluble chloroamide, 1,3,4,6-tetrachloro-3a,6a-diphenylglycoluril. *Biochem. Biophys. Res. Commun.*, **80**, 849-857.

GOLDENBERG, D.A., SHARKEY, R.M. & FORD, E. (1987). Anti-antibody enhancement of iodine-131 anti-CEA radioimmunodetection in experimental and clinical studies. *J. Nucl. Med.*, **28**, 1604-1610.

GREEN, N.M. (1965). A spectrophotometric assay for avidin and biotin based on binding of dyes by avidin. *Biochem. J.*, **94**, 23c-24c.

GREEN, N.M. (1970). Purification of avidin. *Methods Enzymol.*, **18A**, 414-417.

HOFMANN, K., WOOD, S.W., BRINTON, C.C., MONTIBELLER, J.A. & FINN, F.M. (1980). Iminobiotin affinity columns and their application to retrieval of streptavidin. *Proc. Natl Acad. Sci. USA*, **77**, 4666-4668.

KALOFONOS, H.P., RUSCKOWSKI, M., SIEBECKER, D.A., SIVOLAPENKO, G.B., SNOOK, D., LAVENDER, J.P., EPENETOS, A.A. & HNATOWICH, D.J. (1990). Imaging of tumour in patients with indium-111-labeled biotin and streptavidin-conjugated antibodies: preliminary communication. *J. Nucl. Med.*, **31**, 1791-1796.

KEEP, P.A., SEARLE, F., BEGENT, R.H.J., BARRATT, G.M., BODEN, J., BAGSHAWE, K.D. & RYMAN, B.E. (1983). Clearance of injected radioactively labelled antibodies to tumour products by liposome-bound second antibodies. *Oncodevel. Biol. Med.*, **4**, 273-280.

KHAWLI, L.A., ALAUDDIN, M.M., MILLER, G.K. & EPSTEIN, A.L. (1993). Improved immunotargeting of tumors with biotinylated monoclonal antibodies and radiolabeled streptavidin. *Antib. Immunoconj. Radiopharm.*, **6**, 13-27.

LEDERMANN, J.A., BEGENT, R.H.J., BAGSHAWE, K.D., RIGGS, S.J., SEARLE, F., GLASER, M.G., GREEN, A.J. & DALE, R.G. (1988). Repeated antitumour antibody therapy in man with suppression of the host response by Cyclosporin A. *Br. J. Cancer*, **58**, 654-657.

NORRGREN, K., STRAND, S.-E., NILSSON, R., LINDGREN, L. & SJOGREN, H.-O. (1993). A general, extracorporeal immunosorption method to increase the tumor to normal tissue ratio in radioimmunoimaging and radioimmunotherapy. *J. Nucl. Med.*, **34**, 448-454.

PAGANELLI, G., PERVEZ, S., SICCARDI, G., ROWLINSON, G., DELEIDE, G., CHIOLERIO, F., MALCOVATI, M., SCASSELLATI, G.G. & EPENETOS, A.A. (1990*a*). Intraperitoneal radiolocalisation of tumours pre-targeted by biotinylated monoclonal antibodies. *Int. J. Cancer*, **45**, 1184-1189.

PAGANELLI, G., STELLA, M., DE NARDI, P., MAGNANI, P., ZITO, F., SICCARDI, G., DI CARLO, V. & FAZIO, F. (1990*b*). A new method for faster blood clearance in radioimmuno-guided surgery. *J. Nucl. Med. Allied. Sci.*, **35**, 88-89.

PAGANELLI, G., MAGNANI, P., ZITO, F., VILLA, E., SUDATI, F., LOPALCO, L., ROSSETI, L., MALCOVATI, M., CHIOLERIO, F., SECCAMANI, E., SICCARDI, A.G. & FAZIO, F. (1991). Three-step monoclonal antibody tumor targeting in carcinoembryonic antigen-positive patients. *Cancer Res.*, **51**, 5960-5966.

PAGANELLI, G., BELLONI, C., MAGNANI, P., ZITO, F., PASINI, A., SASSI, I., MERONI, M., MARIANI, M., VIGNALI, M., SICCARDI, A.G. & FAZIO, F. (1992). Two-step tumour targeting in ovarian cancer patients using biotinylated monoclonal antibodies and radioactive streptavidin. *Eur. J. Nucl. Med.*, **19**, 322-329.

PEDLEY, R.B., BODEN, J., KEEP, P.A., HARWOOD, P.J., GREEN, A.J. & ROGERS, G.T. (1987). Relationship between tumour size and uptake of radiolabelled anti-CEA in a colonic tumour xenograft. *Eur. J. Nucl. Med.*, **13**, 197-202.

PEDLEY, R.B., DALE, R., BODEN, J.A., BEGENT, R.H.J., KEEP, P. & GREEN, A.J. (1989). The effect of second antibody clearance on the distribution and dosimetry of radiolabelled anti-CEA antibody in a human colonic tumour xenograft model. *Int. J. Cancer*, **43**, 713-718.

PEDLEY, R.B., BEGENT, R.H.J., BODEN, J.A., BODEN, R., ADAM, T. & BAGSHAWE, K.D. (1991). The effect of radiosensitizers on radioimmunotherapy, using $^{131}$I-labelled anti-CEA antibodies in a human colonic xenograft model. *Int. J. Cancer*, **47**, 597-602.

SHARKEY, R.M., PRIMUS, F.J. & GOLDENBERG, D.M. (1984). Second antibody clearance of radiolabeled antibody in cancer radioimmunodetection. *Proc. Natl Acad. Sci. USA*, **81**, 2843-2846.

SHARKEY, R.M., MABUS, J. & GOLDENBERG, D.D. (1988). Factors influencing anti-antibody enhancement of tumor targeting with antibodies in hamsters with human colonic tumor xenografts. *Cancer Res.*, **48**, 2005–2009.

SINITSYN, V.V., MAMONTOVA, A.G., CHECKNEVA, Y.Y., SHNYRA, A.A. & DOMOGATSKY, S.P. (1989). Rapid blood clearance of biotinylated IgG after infusion of avidin. *J. Nucl. Med.*, **30**, 66–69.

TOM, B.H., RUTZKY, L.P., JAKSTYS, M.M., OYASU, R., KAYE, C.I. & KAHAN, B.D. (1976). Human colonic adenocarcinoma cells. *In Vitro*, **12**, 180–191.